# Transcriptional analysis reveals a markedly reduced expression of the voltage-dependent calcium channel α2δ1 subunit in canine prostate cancer compared to benign prostatic hyperplasia

**DOI:** 10.1186/s12917-025-05046-7

**Published:** 2025-10-09

**Authors:** Maciej Zacharski, Maciej Ugorski, Przemysław Prządka, Stanislaw Dzimira, Sonja Škevin, Iwona Sidorkiewicz, Bartłomiej Liszka, Piotr Skrzypczak, Filip Van Nieuwerburgh, Adam Krętowski, Alicja Tomaszek

**Affiliations:** 1 Department of Biochemistry and Molecular Biology, Faculty of Veterinary Medicine, Wroclaw University of Environmental and Life Sciences, Wroclaw, Poland; 2 Department and Clinic of Surgery, Faculty of Veterinary Medicine, Wroclaw University of Environmental and Life Sciences, Wroclaw, Poland; 3 Department of Pathology, Faculty of Veterinary Medicine, Wroclaw University of Environmental and Life Sciences, Wroclaw, Poland; 4 NXTGNT, Faculty of Pharmaceutical Sciences, Ghent University, Ghent, Belgium; 5 Clinical Research Support Centre, Medical University of Bialystok, Bialystok, Poland; 6 Clinical Research Centre, Medical University of Bialystok, Bialystok, Poland; 7 Department of Endocrinology, Diabetology and Internal Medicine, Medical University of Bialystok, Bialystok, Poland

**Keywords:** Canine prostate cancer, Benign prostate hyperplasia, Gene-expression profiling, Calcium channel subunit α2δ1, CACNA2D1

## Abstract

**Background:**

Despite its low incidence, the aggressive nature and high metastatic potential of canine prostate cancer (PC) make it a serious problem in veterinary medicine. However, knowledge of the molecular mechanisms and regulatory networks underlying the development and progression of canine PC is still limited. Using high-throughput methods, many PC-related genes have been identified by comparing gene expression profiles in malignant and non-malignant prostate tissues. In these studies, the reference group containing both normal and hyperplastic prostate tissues specimens was compared to the group composed of cancerous tissues specimens. However, this approach does not allow the exclusion of inflammation-related genes from those directly involved in PC development and progression. Furthermore, the RNA-Seq and microarray data were not validated by additional analyses, including immunohistochemistry (IHC). Therefore, to identify PC-specific genes, a comparative analysis of gene expression profiles in PC and benign prostate hyperplasia (BPH) tissue samples was performed using RNA-Seq, and the results were validated by Nano String technology, Western blotting (WB) and IHC.

**Results:**

Using the NanoString assay, significant differences were identified for 12 genes, of which *ASPM*,* CLU*,* TOP2A*,* TSPAN8/TM4SF3* and *HELLS* were overexpressed in PC compared to BPH, while *ATP6V0A4*,* CACNA2D1*,* CLDN10*,* GOLGA4*,* KLK2*,* NKX3.1* and *SERPINB6* were downregulated in PC. Validation of the differentially expressed genes (DEGs) at the protein level using WB and IHC revealed a highly decreased expression of the voltage-gated calcium channel subunit alpha-2 delta-1 (α2δ1), encoded by the *CACNA2D1* gene, in PC samples compared to BPH.

**Conclusions:**

Our findings make α2δ1 protein a novel and promising diagnostic biomarker to differentiate PC from BPH.

**Supplementary Information:**

The online version contains supplementary material available at 10.1186/s12917-025-05046-7.

This research was funded in part by the National Science Center (Poland) Miniatura 2021/05/X/NZ5/01570.

This work was supported by the Wrocław University of Environmental and Life Sciences (Poland) as part of the research project no. N090/0002/23.

The APC is financed by Wroclaw University of Environmental and Life Sciences.

For the purpose of Open Access, the author has applied a CC-BY public copyright license to any Author Accepted Manuscript (AAM) version arising from this submission.

## Background

Dogs are one of the few species, other than humans, that develop spontaneous prostate cancer (PC) [1]. However, unlike in humans, canine PC is a rare disease, affecting only 0.2–0.6% of dogs [2–4]. It is characterized by late onset, androgen independence, and is generally highly aggressive and metastatic, with the bones and lungs being the primary sites of metastasis [5–9], making it comparable to human castration-resistant prostate cancer (CRPC) [10]. Therefore, given the range of similarities between prostate cancer in dogs and humans, dog represent a relevant model for the human disease [6, 11], and preclinical studies of new diagnostic and therapeutic approaches in dogs may benefit both species affected by prostate cancer.

To date, our knowledge of the molecular mechanisms and regulatory networks underlying the development and progression of canine PC is very limited. Using IHC and the data on differential gene expression between tumor and non-tumor tissues in human PC, only a few changes in the expression of specific markers/proteins have so far been reported in canine PC compared to normal and hyperplastic canine prostate. Similar to findings in humans, decresed expression of Nkx3.1 and overexpression of C-MYC [12, 13], reduced expression of PTEN, p53, and the androgen receptor (AR), as well as increased expression of MDM2 [14, 15], have been observed. Likewise, elevated levels of HER-2, mTOR, and eIF4E proteins have been detected in canine PC compared with normal prostate [16–18]. In addition, loss of membranous E-cadherin, together with cytoplasmic and nuclear β-catenin staining, suggests that activation of the WNT/β-catenin pathway may play a role in the pathogenesis of canine PC. Despite the advantages of this approach, which strongly support the usefulness of canine PC as a model for studying human PC, these studies have a major limitation in that they cannot identify genes specifically involved in the development and progression of canine PC. Such limitations can be overcome using high-throughput methods such as whole-transcriptome RNA sequencing (RNA-Seq) or microarray profiling, which provide information on the expression of thousands of genes simultaneously. RNA-Seq revealed clear differences in gene expression profiles between non-malignant and malignant canine prostate tissue samples [19, 20]. Furthermore, most of these DEGs were mapped to at least one of 49 biological pathways from the Kyoto Encyclopedia of Genes and Genomes (KEGG), suggesting that these pathways are deregulated in canine PC. Based on these data, the DEGs were grouped into five super-pathways named Inflammatory Response and Cytokines, Regulation of Immune System and Cell Death, Cell Cycle, Phagosome and Autophagy, and Cell Surface and PI3K Signaling. With this approach, a set of 16 DEGs representing the most interesting potential biomarkers was proposed, half of which have already been found to be associated with PC [20]. Similarly, using the microarray technique, 33 genes with significantly different expressions between PC and benign prostate tissues were identified [21]. According to this data, AR-regulated genes and neuroendocrine-associated genes were downregulated in cancerous tissues. However, most previous reports lacked specific information on DEGs between PC and BPH tissues, as in these studies both normal and hyperplastic prostatic tissues were combined in the single control group. In the present study, only BPH tissue samples were used for comparison. This approach, aimed at identifying PC-specific genes, has a major advantage: it enables the exclusion of inflammation-related genes. Inflammation is one of the hallmarks of cancer [22, 23], and multiple lines of evidence link the pathogenesis of BPH with prostatic inflammation [24, 25]. Here, we compared the transcriptomes of canine BPH and PC to identify genes and their protein products that may serve as candidate diagnostic biomarkers for distinguishing between these two conditions. As noted above, the current understanding of the molecular mechanisms and regulatory networks underlying the development and progression of canine PC remains limited and is still at an early exploratory stage, particularly in comparison with human PC. Consequently, the identification of canine PC-specific genes presents new opportunities to advance the field through the integration of molecular and cell biology approaches.

## Methods

### Tissue specimens and histochemistry

The study was conducted on prostate specimens collected from 15 dogs with PC and 7 dogs with BPH between 2017 and 2023. Dogs underwent surgery (prostate coagulation, prostatectomy, prostate biopsy, *n* = 12) or euthanasia (*n* = 6) at the Department and Clinic of Surgery, Faculty of Veterinary Medicine, University of Environmental and Life Sciences, Wroclaw, Poland. The remaining samples (*n* = 4) were collected during the necropsy of animals performed within 24 h of the animals’ demise. All owners agreed to the necropsy. Dogs undergoing surgical treatment or euthanasia underwent computed tomography (CT) of the thorax and abdomen to assess the presence of metastatic lesions. According to owner reports, these dogs had no history of chronic systemic diseases and were not receiving any treatment prior to sample collection. Breed, age, neutering status, and TNM classification are presented in Table 1. For the dogs from which material was collected post-mortem, information on neutering status and health history was unavailable, which is a limitation of the study.

**Table 1:** Specimen characteristics

Sample ID	Breed	Age (years)	Neutering Status	Collection Procedure	HPDx	TNM classification	Measurements performed			
							RNA-Seq	NanoString	WB	IHC
Dog 1	N/A	N/A	N/A	necropsy	BPH	N/A	No	Yes	Yes	Yes
Dog 2	N/A	N/A	N/A	necropsy	BPH	N/A	No	Yes	No	Yes
Dog 3	N/A	N/A	N/A	necropsy	BPH	N/A	Yes	Yes	Yes	Yes
Dog 4	N/A	N/A	N/A	necropsy	BPH	N/A	Yes	Yes	Yes	No
Dog 5	Mixed	12	No	surgery	BPH	T0N0M0	Yes	Yes	Yes	Yes
Dog 6	Mixed	8	No	surgery	BPH	T0N0M0	No	Yes	Yes	No
Dog 7	Entlebucher Mount. Dog	7	No	surgery	BPH	T0N0M0	No	Yes	Yes	Yes
Dog 8	Mixed	12	Yes	surgery	PC	T2N1M0	No	Yes	No	No
Dog 9	Mixed	13	Yes	surgery	PC	T1N0M0	No	Yes	Yes	Yes
Dog 10	Mixed	15	Yes	euthanasia	PC	T2N1M1	Yes	Yes	Yes	No
Dog 11	Mixed	-	Yes	euthanasia	PC	T3N1M1	No	Yes	Yes	No
Dog 12	French Bulldog	8	No	euthanasia	PC	T2N1M1	No	Yes	Yes	Yes
Dog 13	Labrador Retriever	10	No	surgery	PC	T2N1M0	No	Yes	No	No
Dog 14	Maltese Dog	10	Yes	surgery	PC	T3N1M1	No	Yes	Yes	No
Dog 15	Mixed	8	Yes	surgery	PC	T1N0M0	No	Yes	Yes	Yes
Dog 16	Mixed	12.5	Yes	surgery	PC	T3N1M0	No	Yes	No	Yes
Dog 17	Mixed	12.5	No	euthanasia	PC	T3N1M0	No	Yes	No	Yes
Dog 18	Staffordshire Bull Terrier	8	No	surgery	PC	T1N0M0	Yes	Yes	No	Yes
Dog 19	Boxer	8	No	surgery	PC	T1N0M0	No	Yes	No	Yes
Dog 20	Mixed	-	No	euthanasia	PC	T3N1M0	No	Yes	No	Yes
Dog 21	Poodle	13	Yes	euthanasia	PC	T3N1M1	No	Yes	No	Yes
Dog 22	Mixed	5.5	Yes	surgery	PC	T1N0M0	No	No	Yes	Yes

*HPDx -* Histopathological Diagnosis, *WB -* Western blotting, *IHC -* immunohistochemistry, *TNM – TNM* Classification of Malignant Tumors

**Table 2:** Results of NanoString gene expression analysis for PC vs. BPH group

Gene	Log_2_FC	*p*-value	adj. *P*-value
**ASPM**	**4.63**	**0.0001**	**0.0001**
**TSPAN8**	**4.59**	**0.0001**	**0.0001**
**TOP2A**	**2.65**	**0.0001**	**0.0001**
**CLU**	**2.18**	**0.0013**	**0.0026**
**HELLS**	**1.69**	**0.0026**	**0.0050**
COL6A3	1.38	0.0359	0.0643
CHI3L1	1.26	0.0877	0.1176
IGFBP2	1.25	0.0603	0.0932
MSH6	1.21	0.0727	0.1075
SULF2	1.18	0.0553	0.0895
MSH2	1.12	0.0899	0.1176
IGFBP5	0.70	0.3107	0.3522
SMOC2	0.32	0.6420	0.6615
FBN2	0.30	0.7353	0.7353
SGO2	−0.26	0.5242	0.5570
CENPF	−0.42	0.4012	0.4400
CD302	−0.96	0.0871	0.1176
NPTX1	−1.71	0.1296	0.1632
**GOLGA4**	**−2.40**	**0.0001**	**0.0002**
GLI1	−2.56	0.0380	0.0646
**SERPINB6**	**−2.61**	**0.0001**	**0.0001**
**CACNA2D1**	**−6.09**	**0.0001**	**0.0001**
**ATP6V0A4**	**−7.86**	**0.0001**	**0.0001**
**CLDN10**	**−8.02**	**0.0001**	**0.0003**
**NKX3-1**	**−8.22**	**0.0001**	**0.0001**
**KLK2**	**−12.73**	**0.0001**	**0.0001**

Bolded font marks genes showing statistically significant differential expression

**Table 3:** Semiquantitative assessment of α2δ1 immunohistochemical labeling in the canine prostate gland with prostate cancer (PC) and benign prostatic hyperplasia (BPH)

Sample ID	Histopathological diagnosis	Distribution	Staining intensity
Dog 1	*BPH*	absent	0
Dog 2	*BPH*	very low	+
Dog 3	*BPH*	absent	0
Dog 5	*BPH*	moderate	+++
Dog 7	*BPH*	moderate	++
Dog 9	*PC*	very low	+
Dog 12	*PC*	very low	0/+
Dog 15	*PC*	absent	0
Dog 16	*PC*	absent	0
Dog 17	*PC*	low	+/++
Dog 18	*PC*	very low	+
Dog 19	*PC*	absent	0
Dog 20	*PC*	very low	+
Dog 21	*PC*	absent	0
Dog 22	*PC*	absent	0

Samples were grouped in 6 categories based on the number of cells positive for α2δ1 expression: absent (0% labelled cells), very low (1–5% labelled cells), low (6–20% labelled cells), moderate (21–50% labelled cells), high (51–60% labelled cells), very high (> 60% labelled cells). Staining intensity: (0) absent, (+) weak, (++) moderate, (+++) intense

For the histochemistry, the Material was fixed in buffered 7% formalin and embedded in paraffin. The 3-µm-thick cut sections were mounted on Superfrost Plus slides, deparaffinized in xylene, and rehydrated to distilled water. The samples were then incubated in Mayer’s hematoxylin for 3 min and washed with three changes of tap water or until blue stopped coming off the slides. Following this, the slides were counterstained in alcoholic eosin for 10 s without rinsing, dehydrated through two changes of 95% ethanol, two changes of 100% ethanol, two changes of acetone, and cleared in two changes of xylene (10 s each). Finally, the slides were mounted with cover slides [26].

### RNA isolation

Total RNA was purified from 30 milligrams of tissue using the RNeasy Fibrous Tissue Mini Kit (Qiagen) according to the manufacturer’s instructions. The protocol included on-column DNase digestion to remove genomic DNA. The purity and concentration of the RNA were assessed using a DS-11 FX spectrophotometer (Denovix), and its quality was assessed by Experion microchip electrophoresis (Biorad).

All RNA samples met the quality control thresholds required for downstream analysis. Samples used for RNA-Seq had a mean RNA Integrity Number (RIN) of 8.4 (range: 7.6–9.9) and a mean concentration of 723 µg/µl (range: 188–1182 µg/µl). For the NanoString assay, the mean RIN was 8.0 (range: 5.4–9.6) with a mean concentration of 1064.4 µg/µl (range: 118–2063 µg/µl) (Additional file 1. Supplementary Fig. 1).

### RNA sequencing

The library was prepared using 350 ng of total RNA and the QuantSeq 3’ mRNA library preparation FWD kit (Lexogen) according to the manufacturer’s instructions. Briefly, the first strand of cDNA was synthesized, RNA was removed, and the second strand was synthesized using Unique Molecular Identifiers (UMI). Library preparation encompassed 13 cycles of PCR enrichment (initial denaturation: 98 °C, 30 s.; 13 cycles of 98 °C for 10 s, 65 °C for 20 s, 72 °C for 30 s; final elongation: 72 °C, 1 min) followed by purification of the amplified product. Subsequent to this, a quality control assessment of the libraries was conducted using the Bioanalyser High Sensitivity DNA chip (Agilent Technologies). Thereafter, the libraries were quantitatively assessed by qPCR in accordance with the Illumina protocol. This facilitated the equimolar pooling of the libraries, which were subsequently sequenced on the NextSeq500 (Illumina) instrument using single reads of 75 base pairs on a high-output configuration.

Reads trimmed and filtered with Trimmomatic [27] were mapped to the *C. lupus familiaris* genome version CanFam3.1, annotation release 101, (GeneBank accession: GCF_000002285.3) using STAR v2.7.6a aligner [28]. RSEM v1.2.28 [29] was used to calculate gene count with –rsem-calculate-expression option. The gene counts were made into a count table in R, where genes with no count and low count genes were filtered out. EdgeR v3.30.3 [30] package was used for normalization, calculating dispersion and testing for differential expression.

### Targeted mRNA profiling

To evaluate the transcriptomic profiles custom NanoString CodeSet (NanoString Technologies) was utilized. Total RNA was extracted from fresh frozen samples using the RNeasy Fibrous Tissue Mini Kit (Qiagen). RNA samples were prepared by hybridization (65 °C) to nCounter Reporter and Capture probes according to the Manufacturer protocol. Subsequent to the automated sample cleanup, reporter capture, and alignment on the cartridge by the nCounter Prep Station, the RNA samples were deemed ready for analysis. The sample cartridges were then placed in the nCounter Digital Analyzer for data collection. The study incorporated 555 field-of-view readings, and nSolver 4.0.7 analysis software (NanoString) was employed for data analysis. This software included background correction (20 counts were designated as the background threshold) and normalization using both positive control and five reference (housekeeping) genes. *P*-values were adjusted to account for multiple testing using the Benjamini-Hochberg false discovery rate (FDR) method and adjusted p-value ≤ 0.05 was considered significant. The threshold value for significance was set at a fold change (FC) of ≥ 1.5 to identify DEGs.

The NanoString normalized data were calculated using the Rosalind web-based software with the settings described above for nSolver 4.0.7 analysis software (NanoString Technologies). For the calculation of the Z-values and creation of the gene expression heatmap, normalized expression data and the Heatmapper web-based tool was used [31]. The genes and samples were clustered using average linkage and Pearson Distance Measurement Method.

### Western blotting

Tissue samples, weighing 30 milligrams, were homogenized in 350 µl of ice-cold RIPA buffer (50 mM Tris-HCl, 150 mM NaCl, 1.0% NP-40, pH 8.0) containing Halt Protease Inhibitor Coctail (Thermo-Fisher). The resulting mixture was then subjected to a 30-minute incubation on ice. Subsequently, the samples were subjected to a centrifugal process at 16,000 × g for 20 min at a temperature of 4 °C. The resultant clear upper layer was collected and stored on ice. Protein quantification was performed using the BCA assay (Pierce) according to the manufacturer’s instructions.

Protein samples (30 µg) were prepared by mixing with 5x Laemmli buffer (containing 15% 2-mercaptoethanol) and subsequently denatured at 95 °C for five minutes. Following this step, the samples were allowed to cool on ice, and then loaded onto mini-gels prepared with 7.5% or 12% TGX Stain-free Fast Cast Kit (Bio-Rad). Electrophoresis was conducted at 120 V. After 45 s UV-activation of the gel’s Stain-free chemistry, proteins were transferred to a PVDF membrane at 350 mA for 60 min, blocked for 1 h in blocking buffer (5% skimmed milk, 0.05% Tween-20 in PBS) and then incubated with primary antibodies diluted in the blocking buffer (mouse-raised monoclonal antibody anti-voltage-gated calcium channel subunit α2δ1, Thermo-Fisher, cat.no MA3-921; rabbit monoclonal antibody anti-TOP2A, CST, cat. no. 12286 T; rabbit polyclonal antibody anti-GOLGA4, CST, cat. no. 79145 S; rabbit polyclonal antibodies anti-CLDN10, Abclonal, cat. no. A9853) overnight at 4 °C. The following day, the membrane was washed, and incubated for 1 h at room temperature with anti-mouse HRP-conjugated antibody (Jackson ImmunoResearch, cat. no. 115-035-003, diluted 10000x) for α2δ1 detection, or with a goat polyclonal anti-rabbit HRP-conjugated antibody (DAKO, cat.no P044801-2, diluted 5000x) for detection of the remaining targets. Secondary antibodies were diluted in the blocking buffer. Following a thorough washing step, total protein was subjected to visualization through the implementation of the Stain-free chemistry method. The subsequent imaging of target-specific bioluminescent signals was conducted utilizing the SuperSignal West Femto ECL substrate (Pierce) and the Chemidoc Touch (Bio-Rad) imaging system. The densitometry analysis was performed by normalizing the bioluminescent signals against the total protein stain-free signal from the corresponding lane of the blot.

### Immunohistochemistry

For IHC, FFPE tissues were freshly cut into 3-µm-thick sections and mounted on Superfrost Plus slides (Menzel Gläser). Staining was then performed on a LEICA BOND-MAX (Leica Biosystems) according to the following protocol. Initially, the tissues were deparaffinized using Bond Dewax Solution (Leica Biosystems) and then pre-treated with Bond Epitope Retrieval Solution 1 (Leica Biosystems) for a period of 20 minutes. The activity of the endogenous peroxidase was blocked by Peroxide Block using the BOND Polymer Refine Detection System (Leica Biosystems). Rabbit polyclonal antibodies against α2δ1 were purchased from ABclonal (cat.no A15260). The antibodies were diluted 1:100 with Bond Primary Antibody Diluent (Leica Biosystems) and sections were incubated with primary antibodies for 15 minutes at RT. The samples were then incubated with post-primary and polymer using the BOND Polymer Refine Detection System (Leica Biosystems). The reaction substrate was 3,3’-diaminobenzidine (DAB chromogen) and all sections were counterstained with hematoxylin (BOND Polymer Refine Detection System, Leica Biosystems). Control experiments were performed in the absence of primary antibody. The results of the immunohistochemical reaction were scored within each sample using a semiquantitative scoring system (0 - no staining, + (1) - low, ++ (2) - moderate, +++ (3) - high), taking into account the intensity of cell staining.

### Statistical analysis

Statistical analysis of the RNA-Seq and NanoString data is described in their respective sections of the Methods. Data normality was assessed using the Shapiro-Wilk test. To evaluate differences in mean values for the Western Blot densitometry and IHC data between the BPH and PC groups, the Mann-Whitney U test was used. A *P*-value < 0.05 was considered statistically significant.

## Results

Each tissue specimen was divided into three parts: fresh frozen, RNA-later-treated frozen and formalin-fixed paraffin-embedded (FFPE). RNA-later–treated frozen tissue samples from dogs 3, 4, 5, 10, and 18 were used for RNA-seq, and all such samples, except one, were used for NanoString; fresh frozen tissue samples from 13 dogs (1, 3, 4, 5, 6, 7, 9, 10, 11, 12, 14, 15, 22) were used for WB; FFPE sections from 15 dogs (1, 2, 3, 5, 7, 9, 12, 15–22) were used for IHC (Table 1). Sample selection criteria differed between the initial transcriptomic profiling and subsequent experiments. For the RNA-Seq analysis, conducted on a preliminary set of samples, specimens with the highest RIN were chosen. Following the completion of sample collection, tissues for NanoString, WB, and IHC validation were selected based on availability.

###  Comparative molecular profiling of prostate carcinoma and benign prostate hyperplasia

**Fig. 1:**
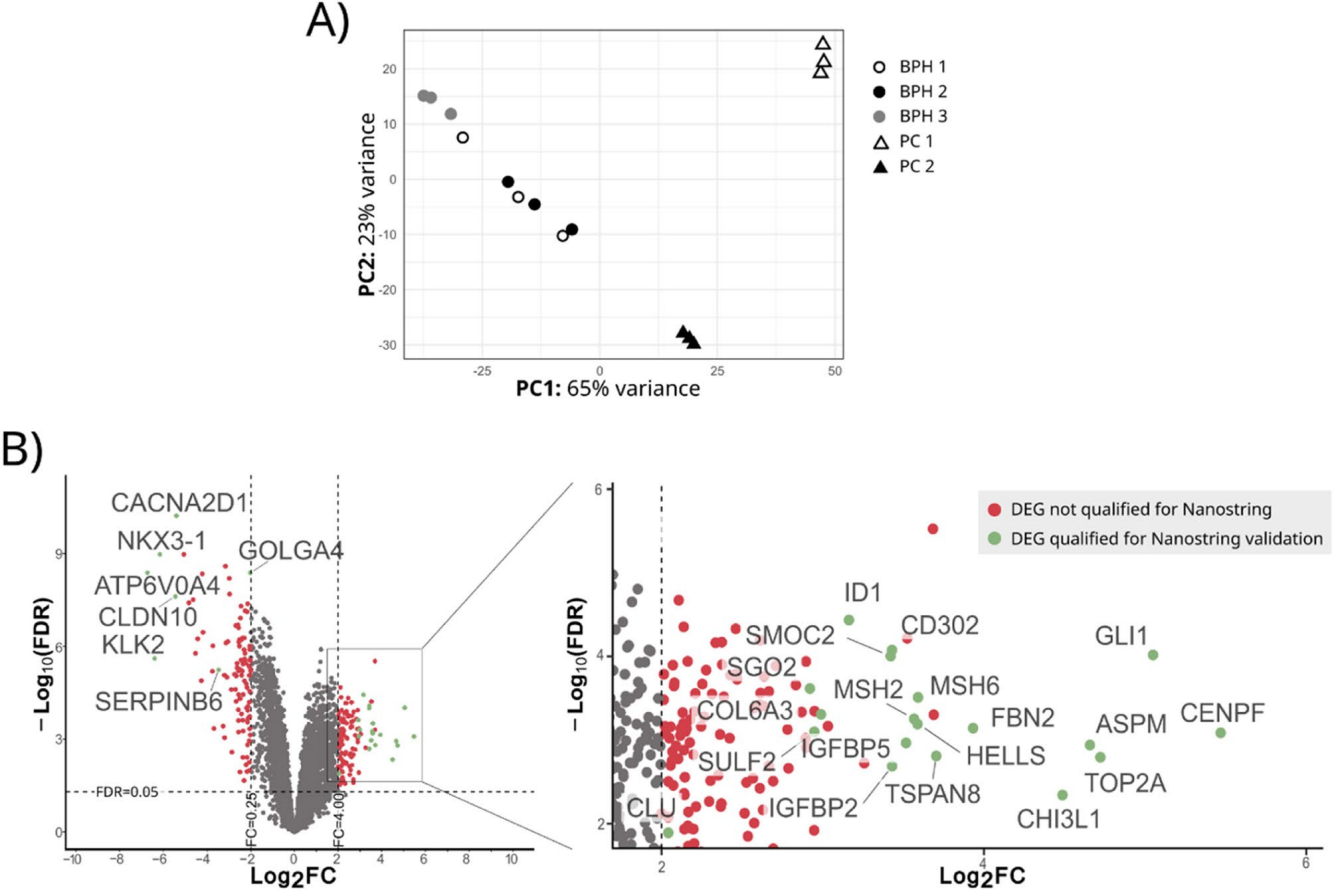
Differentially expressed genes (DEGs) between prostate cancer (PC) and benign prostatic hyperplasia (BPH). **A**) Principal component analysis (PCA) was employed to demonstrate the differences between PC (triangle) and BPH (circle) tissue samples, as well as the variances between replicates of individual samples. PCA elucidated that the majority of the variance (65%) was attributable to the histopathological typing of tissue specimens. **B)** Volcano plot showing highly differentially expressed genes (DEGs) in PC vs. BPH (−2>log_2_FC>2, FDR>0.05), mean normalized counts per million (CPM) >10 in each group. DEGs in green were selected for validation by targeted mRNA profiling (NanoString CodeSet) based on the highest differences in expression and presence of mRNA in both samples (PC and BPH). For clarity, an enlarged section of the volcano plot shows the DEGs upregulated in PC.

 RNA-Seq data from two PC tissues and three BPH tissues subjected to bioinformatic analysis revealed 2923 DEGs out of a total of 12 185 genes identified in the studied samples (Additional file 1, Supplementary Figure 2). After subjecting the RNA-Seq data to principal component analysis (PCA), it was established that the substantial disparities (65%) in transcriptomic profiles between PC and BPH samples were in congruence with the histopathological evaluation of the corresponding tissue specimens (Fig. 1A). Nevertheless, PCA also revealed heightened heterogenicity between PC samples as opposed to BPH samples. Among the DEGs exhibiting at least a minimum expression level in both groups, 114 genes demonstrated significant upregulation, while 92 genes exhibited significant downregulation in the PC samples compared to the BPH specimens. These genes were selected based on a log_2_-fold change (log_2_FC) less than −2 or greater than 2, the FDR less than 0.05, and a mean normalized counts per million (CPM) greater than 10 in each group (Fig. 1B; Additional file 2. Supplementary Table 1).

**Fig. 2:**
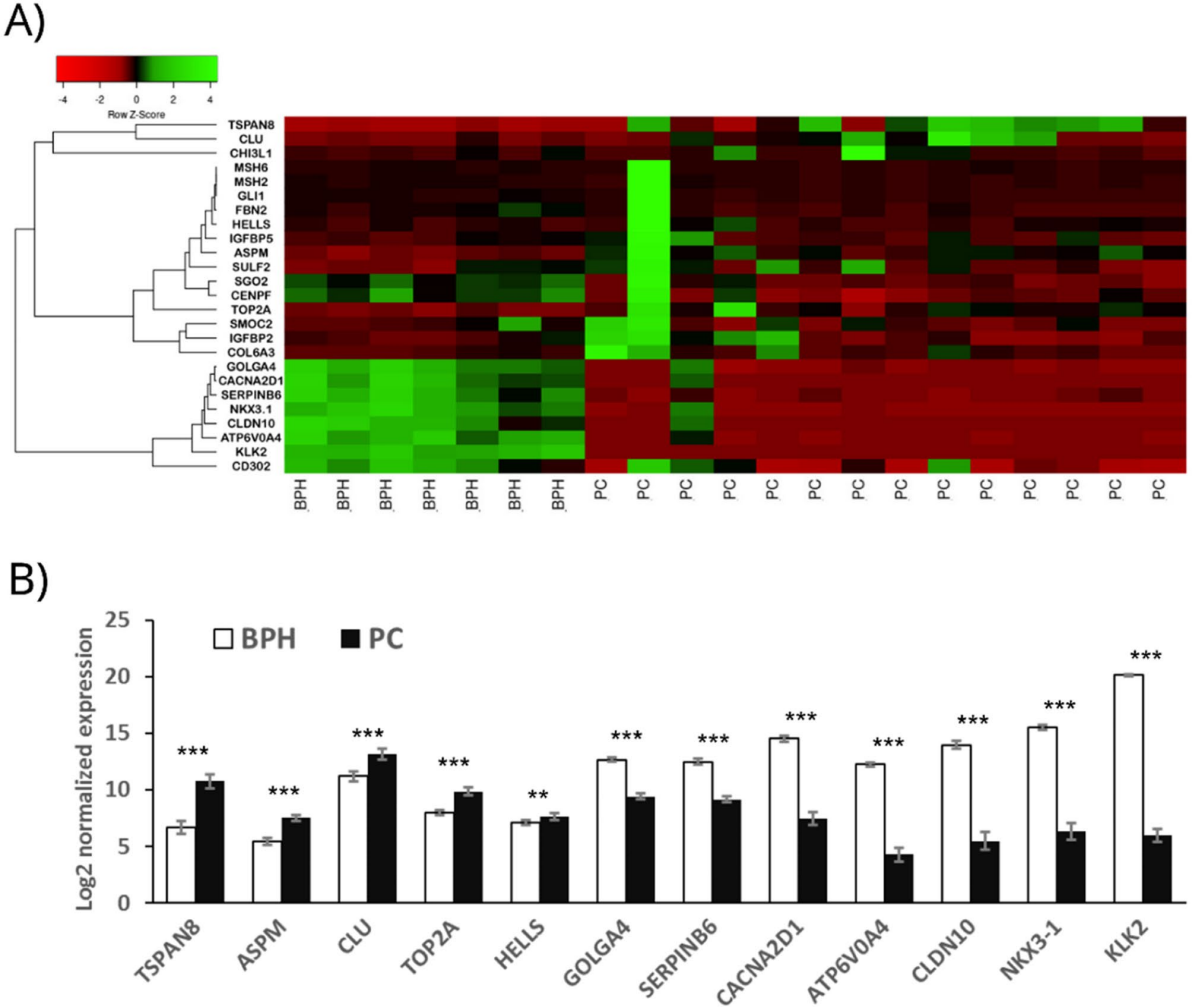
Differentially expressed genes (DEGs) between prostate cancer (PC) and benign prostate hyperplasia (BPH) validated by targeted mRNA profiling using the NanoString assay. **A)** Heatmap representing Z-score based on the normalized gene expression of all NanoString-analyzed genes shows clusters of genes with similar expression patterns. It indicates that the gene expression profiles of *TSPAN8* and *CLU* are closely related and distinct from those of CACNA*2D1* and *GOLGA*, which constitute a separate cluster. **B)** The mean log2-normalized expression level of genes that exhibited statistically significant differences between the BPH and PC groups (**p< 0.01, *** p< 0.001). The DEGs were sorted from those with the highest expression levels in the PC compared to BPH. Error bars represent SEM.

In the next step, 19 upregulated and 7 downregulated DEGs were selected to validate the RNA-Seq data using the NanoString technology on a larger (n = 14 PC, n = 7 BPH) patient cohort (Figure 1B; Additional file 2, Supplementary Table 1). In most cases, this selection was based on the highest FC, but based on the literature data some of the genes were excluded (*SELP*, *RGS4*, *MATN2*, *MATN4*, *CHAF1A*, *NUP107*, *ATF3*), and some, with lower FC, were added to the validation set (*CLU*, *GOLGA4*, *SERPINB6*). The total RNA isolated from fresh frozen tissue samples was subjected to PCR-free targeted mRNA profiling by Direct Digital Detection. The complete results are presented as a heatmap of normalized gene expression (Figure 2A). The cluster consisting of PC overexpressed genes was further divided into two subclusters. The three genes highly upregulated in PC samples compared to BPH, *TSPAN8*, *CLU* and *CHI3L1*, represented the first sub-cluster. Whereas the remaining genes (*ASPM, TOP2A, HELLS, COL6A3, IGFBP2, MSH6, SULF2, MSH2, IGFBP5, SMOC2, FBN2, SGO2, CENPF, GLI1*), representing the second sub-cluster, did not show such robust differences between the study groups. The cluster of PC underexpressed genes contained 8 genes *(NKX3.1, CLDN10, ATP6V0A4, KLK2, CD303*, *GOLGA4A*, *CACNA2D1* and *SERPINB6*), among which the last three showed the most similar expression pattern. Based on NanoString assay, we found statistically significant differences in the expression of the following 12 genes: *ASPM, ATP6V0A4, CACNA2D1*, *CLDN10*, *CLU, GOLGA4*, *HELLS*, *KLK2, NKX3.1*, *SERPINB6*, *TOP2A*, and *TSPAN8* (Figure 2B, Table 2). Five of these genes: *TSPAN8*, *ASPM*, *CLU*, *TOP2A*, and *HELLS* were overexpressed in PC compared to BPH, while the remaining were downregulated in PC.

### Validation of selected DEG expression on the protein level

**Fig. 3:**
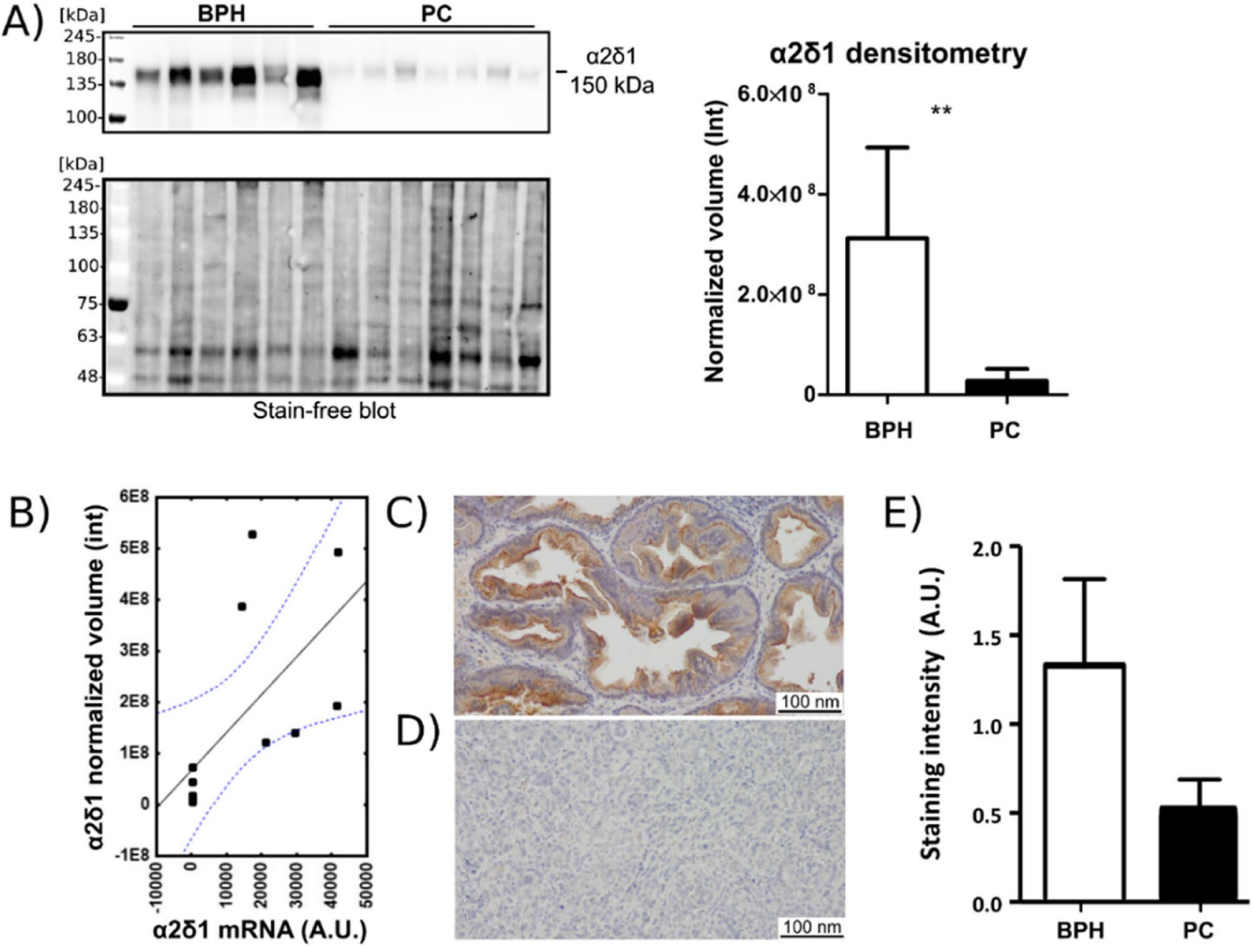
Expression of the voltage-gated calcium channel subunit α2δ1 (α2δ1) in PC and BPH tissue specimens. **A)**Western blotting analysis of anti-α2δ1 murine monoclonal antibody binding to proteins of PC and BPH tissues. Tissue lysates equivalent to 30 µg of protein were separated by SDS-PAGE under reducing conditions on a 7.5% gel and electrophoretically transferred onto a PVDF membrane. The densitometry analysis was performed by normalizing the bioluminescent signals against the total protein stain-free signal from the corresponding lane of the blot. Band intensities were analyzed with Image Lab software version 6.0.0 build 25 (Bio-Rad Laboratories) and GraphPad Prism version 5. (**p< 0.01). Full-length blots are presented in Additional file 1, Supplementary Figure 3. **B) **Positive correlation between intensity of α2δ1 protein expression (Western blotting) and α2δ1 mRNA expression (NanoString) in BPH. r_s_= 0.81;*p*= 0.0014. **C) **Immunohistochemical staining of BPH and PC **(D) **with anti-α2δ1 rabbit polyclonal antibodies. **E)** Reaction intensities with anti-α2δ1 rabbit polyclonal antibodies were calculated on the basis of a semiquantitative scoring system [0- no staining, + (1) - low, ++ (2) - moderate, +++ (3) – high]. Error bars represent SEM.

In order to provide further validation of the data on protein level, the expression of DEGs was analyzed employing WB and IHC. The targets for protein-level validation via immunochemical methods were selected from the set of NanoString-confirmed DEGs based on the commercial availability of antibodies. The qualified antibodies were directed against human antigenic epitopes which share over 90% sequence homology with canine epitopes and preferably were monoclonal. Therefore, the following DEGs were subjected to analysis by WB: *TOP2*, *GOLGA4*, *CLDN10*, and *CACNA2D1*. Despite the availability of antibodies, the NKX3.1 protein and kallikrein-related peptidase 2 (encoded by the *KLK2* gene) were excluded from WB analysis due to their well-documented association with PC. WB analysis using mouse monoclonal antibodies directed against voltage-dependent α2δ1 revealed the presence of an intense band with an apparent molecular Mass of approximately 150 kDa in tissue lysates from BPH, which was barely detectable in PC protein extracts. (Figure 3 A). Densitometric analysis showed that the mean normalized volume of the α2δ1 band is 11 times lower in PC than in BPH and the difference is statistically significant. Spearman’s correlation analysis shows a strong significant positive correlation between the expression of α2δ1 protein and mRNA (r_s_= 0.81; *p*= 0.0014) (Figure 3 A). These findings were further confirmed by IHC. The expression of α2δ1 in paraffin sections of PC and BPH tissue samples was analyzed using the rabbit polyclonal antibodies. On average, PC samples stained weaker with the anti-α2δ1 antibody than the BPH samples (Figure 3B, Table 3). No binding to canine proteins was observed in the case of antibodies directed against DNA topoisomerase IIα (coded by *TOP2A*), Golgin subfamily A member/GOLGA4 protein (coded by *GOLGA4*), and claudin-10 (coded by *CLDN10*).

## Discussion

 In the case of canine PC, knowledge of its molecular mechanisms and regulatory networks is mostly derived from comparative studies and is limited to changes in the expression of a few genes/proteins known from studies of human PC pathogenesis [32]. These limitations could be overcome using high-throughput methods, but screening of PC tissue versus benign prostate tissue using such an approach, involving the analysis of thousands of genes, has only been performed a few times, and none of these reports supported their findings with a protein-level data. Thiemeyer et al. [20] used RNA-Seq to compare gene expression profiles in malignant and non-malignant prostate tissues to identify potential biomarkers associated with the development and progression of canine PC. As many as 4098 DEGs were found between PC and benign prostate samples, and 688 proteins encoded by these genes were associated with specific biological processes, such as inflammatory response and cytokines, regulation of the immune system and cell death, cell cycle, phagosome and autophagy, and cell surface and PI3K signaling, which are known to be highly deregulated in cancer in general. These cellular processes were described by the authors as “hallmarks of canine PC”. Taking into account their own RNA-Seq data and information from databases and literature on PC-associated genes, the following 16 DEGs were selected as the most promising potential biomarkers of canine PC: *AR, CDKN1A, CTNNB1, EGFR, KLK2, NKX3.1, PDGFRA, SRD5A2, CREB3L4, PDGFRB, NRAS, PIK3CD, PIK3CG, KLK4, KIT,* and *ABCB1.* It should be pointed out that majority of these canine PC-associated genes (13) are also deregulated in human PC (*SRD5A2, KLK2, NKX3-1, CREB3L4, EGFR, PDGFRA, PDGFRB, NRAS, PIK3CD, CTNNB1, PIK3CG,* and *CDKN1A*) and are used in commercial PC multi-gene diagnostic assays. This study was performed using PC specimens from intact as well as neutered dogs. Another study used only the castrated dog cohort to compare gene expression profiles between PC and benign prostate tissue specimens by microarray analysis [21]. A set of 33 genes was identified that showed significantly different expression levels between cancerous and benign tissues. Since canine PC resembles human CRPC in many ways, their gene expression profiles were compared, and five of these genes - *ISG15, AZGP1, GPX3, S100P, and IFITM1*- also showed similar differences in expression compared to human prostate tissue. Both studies confirmed that canine and human PC share many similarities not only clinically but also at the molecular level, but the gene expression profiles were very different, with only two common genes: *KLK2* and *NKX3.2.* It should be noted that in both cases the mRNAs isolated from cancerous tissues were compared with the mixture of mRNAs purified from normal as well as hyperplastic prostate tissues, and it should be emphasized that several lines of evidence link the pathogenesis of BPH with prostatic inflammation [24, 25]. Since inflammation is one of the hallmarks of cancer [22, 33], this approach does not allow us to clearly separate inflammation-related genes from those directly involved in the development and progression of PC. Moreover, previous studies did not validate RNA-Seq and microarray data with complementary analyses. Therefore, in this study only BPH tissues were used to identify PC-specific genes using RNA-Seq. It should be noted that the QuantSeq method employed here sequences only the 3′ end of polyadenylated RNAs, which makes it cost-effective and suitable for degraded samples. However, in contexts different from ours, this approach has serious limitations, as it does not allow the reconstruction of full-length mRNA sequences or the detection of alternative splicing events and gene fusions, which can be identified using standard RNA-Seq. Finally, the selected DEGs were validated by NanoString, Western blotting, and IHC. A comprehensive analysis of the RNA-Seq data revealed a significant upregulation of 114 genes and a significant downregulation of 92 genes in PC samples compared to BPH samples. To validate the findings, the RNA-Seq data underwent additional rigorous bioinformatic analysis, which enabled the selection of 26 genes with the most significant FC between PC and BPH tissues for analysis by NanoString. Thereby, significant differences were identified for the following 12 genes: *ASPM, ATP6V0A4, CACNA2D1, CLDN10, CLU, GOLGA4, HELLS, KLK2, NKX3.1, SERPINB6, TOP2A,* and *TSPAN8.* Five of these genes: *TSPAN8, ASPM, CLU, TOP2A*, and *HELLS* were overexpressed in PC compared to BPH, while the rest of them were downregulated in PC. When these results were compared with previous transcriptomic data [20, 21] two of these genes: *KLK2* and *NKX3.1* were also found to be downregulated in PC compared to non-malignant prostate tissues in both analyses, which strongly indicates that their suppressed expression is the hallmark of canine PC. It is worth noting that differences in the expression of *KLK2* and *NKX3.1* genes between malignant and non-malignant tissues were confirmed at the protein level using IHC and Western blotting [12, 13, 34]. *KLK2* encodes kallikrein-related peptidase (Klk2) and, in contrast to dogs, its expression in humans is higher in PC than in benign prostate tissue, where it is recognized as a biomarker associated with prostate cancer risk [35, 36]. In humans, Klk2 promotes PC cell growth and is involved in tumor metastasis [37]; however, in canine PC, its role remains completely unknown. The expression of Nkx3.1, the product of a prostate-specific tumor suppressor gene, is also markedly decreased in human PC [38, 39]. This transcription factor plays a key role in normal prostate development and tumor suppression as a negative regulator of epithelial cell growth in prostate tissue [40, 41]. However, its specific role in the development and progression of canine PC has never been investigated in experimental studies using cellular models. In addition to these genes, the downregulation of two other genes, *CLDN10* and *CACNA2D1*, was also observed in transcriptomic studies, but this finding has not been validated at the protein level [21]. 

Here, after establishing the statistical significance of 12 DEGs via Nanostring analysis, we performed validation for select genes' protein products using WB and IHC: However, protein-level validation of the DEGs using specific antibodies revealed only a markedly reduced expression of the voltage-gated calcium channel subunit α2δ1, encoded by the *CACNA2D1* gene, in PC samples compared to BPH. Antibodies directed against human TOP2, GOLGA4, and CLDN10 proteins did not bind to the canine ortologs. This is likely due to the absence of cross-reactivity with canine proteins, which highlights the challenges associated with the limited availability of species-specific antibodies targeting canine proteins. At the research level, this issue can be addressed by employing alternative approaches such as mass spectrometry, particularly in combination with liquid chromatography (LC/MS/MS). Nevertheless, given the complexity of this technique, which requires specialized equipment and expertise, it cannot replace immunological methods in routine veterinary diagnostics. 

Little is known about the role of the α2δ1 subunit of the calcium channel in the development and progression of cancer. To date, α2δ1 has been shown to be involved in the self-renewal and tumorigenicity of human hepatocellular carcinoma (HCC) tumor-initiating cells (TICs) [42] and recognized as a TIC marker in non-small cell lung carcinoma [23]. HCC TICs with high expression of α2δ1 were also more invasive and resistant to apoptosis than their α2δ1-negative counterparts. At the molecular level, α2δ1 isoform 5 was found to play a key role in modulating calcium influx as a subunit of L- and N-type voltage-gated calcium channels, which in turn activates the ERK1/2 signaling pathway. Therefore, in the case of human HCC, voltage-gated calcium channel subunit α2δ1 seems to play an important role in its development and progression/recurrence, acting as an oncogenic protein. Interestingly, in the case of canine prostate cancer cells, downregulation of *CACNA2D1* gene expression suggests the suppressive role of α2δ1 in PC development and progression. Further studies using cellular models are needed to answer this question.

## Conclusions

It should be emphasized that dogs with PC or BPH, in contrast to healthy dogs, may present in the clinic with similar clinical signs, such as straining to urinate, pollakiuria, hematuria, or constipation, which greatly hinders and often prevents distinguishing between these two conditions. Therefore, our findings identify the α2δ1 subunit as a novel and promising diagnostic biomarker for differentiating PC from BPH. Considering the availability of aspirates collected by ultrasound-guided fine-needle aspiration (US-FNA) biopsy or less invasive prostatic catheter biopsy of the canine urethra, which are used in the diagnosis of various canine prostatic diseases through different diagnostic approaches [43] e.g., flow cytometric phenotyping [44], further studies are warranted to confirm the usefulness of this biomarker in the differential diagnosis of PC and BPH. To build upon these findings and confirm the biomarker's utility across diverse datasets, the field would benefit from a large-scale meta-analysis. However, we stress that such a study would require a dedicated, harmonized reprocessing of all available raw data to properly account for technical confounders. Last but not least, the role of α2δ1 in prostate cancer biology remains unclear and requires further study using prostate cancer cell line models. 

## Supplementary Information

Additional File 1. Supplementary Figure 1. Scatter plot showing RNA integrity number and RNA concentration of the samples used in NanoString profiling. Supplementary Figure 2. Volcano plot showing 2923 DEGs in PC vs. BPH (-1>log_2_FC>1, FDR>0.05) out of 12185 identified genes. After additional filtering (mean normalized CPM >10 in each group), DEGs in green were selected for validation by targeted mRNA profiling (NanoString technology) based on the highest differences in expression and presence of mRNA in both samples (PC and BPH). For clarity, an enlarged section of the volcano plot shows the DEGs upregulated in PC. Supplementary Figure 3. A) Full-length blot presented in Fig.3A, and B) corresponding Stain-Free loading control. C) Blot replicate used together with A) for densitometric analysis presented in Fig. 3B. and D) corresponding Stain-Free loading control.

Additional File 2. Supplementary Table 1. Results of RNA-Seq differential gene expression analysis for PC vs BPH group. These genes were selected based on a log2-fold change (log2FC) less than -2 or greater than 2, a FDR greater than 0.05, and a mean normalized CPM greater than 10 in both groups. List of the DEGs. The names of the genes selected for NanoString validation were bolded.

## Data Availability

RNA-Seq and NanoString data were deposited into the Gene Expression Omnibus database under accession numbers GSE299084 and GSE299093, respectively. These data are available at the following URLs: [https://www.ncbi.nlm.nih.gov/geo/query/acc.cgi? acc=GSE299084](https:/www.ncbi.nlm.nih.gov/geo/query/acc.cgi? acc=GSE299084),[https://www.ncbi.nlm.nih.gov/geo/query/acc.cgi? acc=GSE299093](https:/www.ncbi.nlm.nih.gov/geo/query/acc.cgi? acc=GSE299093). The remaining data supporting the conclusions of this article are included within the article, and additional files, and are available from the corresponding author upon reasonable request.

## Abbreviations

α2δ1: Voltage–gated calcium channel alpha–2 delta–1 subunit
AR: Androgen receptor
BPH: Benign prostatic hyperplasia
CPM: Counts per million
CRPC: Castration–resistant prostate cancer
DAB: 3,3’–diaminobenzidine
DEG: Differentially expressed gene
FC: Fold change
FDR: False discovery rate
FFPE: Formalin–fixed paraffin–embedded
HPDx: Histopathological Diagnosis
HCC: Human hepatocellular carcinoma
IHC: Immunohistochemistry
KEGG: Kyoto Encyclopedia of Genes and Genomes
PC: Prostate cancer
PCA: Principal component analysis
PIA: Prostatic inflammatory atrophy
RNA: Seq–RNA sequencing
TIC: Tumor–initiating cell
TNM: Tumor Nodes Metastasis Classification of Malignant Tumors
UMI: Unique molecular identifiers
WB: Western blotting
