# Supplementary material for: Transcriptional analysis reveals a markedly reduced expression of the voltage-dependent calcium channel α2δ1 subunit in canine prostate cancer compared to benign prostatic hyperplasia
Source: BMC Vet Res. 2025 Oct 9;21:593. doi: 10.1186/s12917-025-05046-7 (PMC12512813; doi:10.1186/s12917-025-05046-7)
Supplement: Supplementary file 2 — Additional File 2. Supplementary Table 1. Results of RNA-Seq differential gene expression analysis for PC vs BPH group. These genes were selected based on a log2-fold change (log2FC) less than -2 or greater than 2, a FDR greater than 0.05, and a mean normalized CPM greater than 10 in both groups. List of the DEGs. The names of the genes selected for NanoString validation were bolded. [file 12917_2025_5046_MOESM2_ESM.docx]

Additional file 1

**
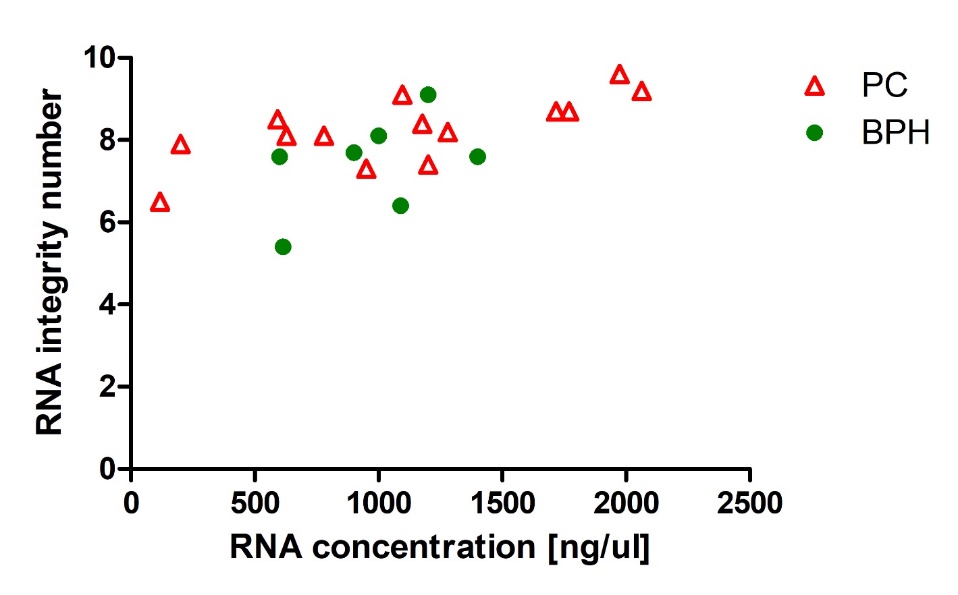
**

**Supplementary Figure 1. Scatter plot showing RNA integrity number and RNA concentration of the samples used in Nanostring profiling.**


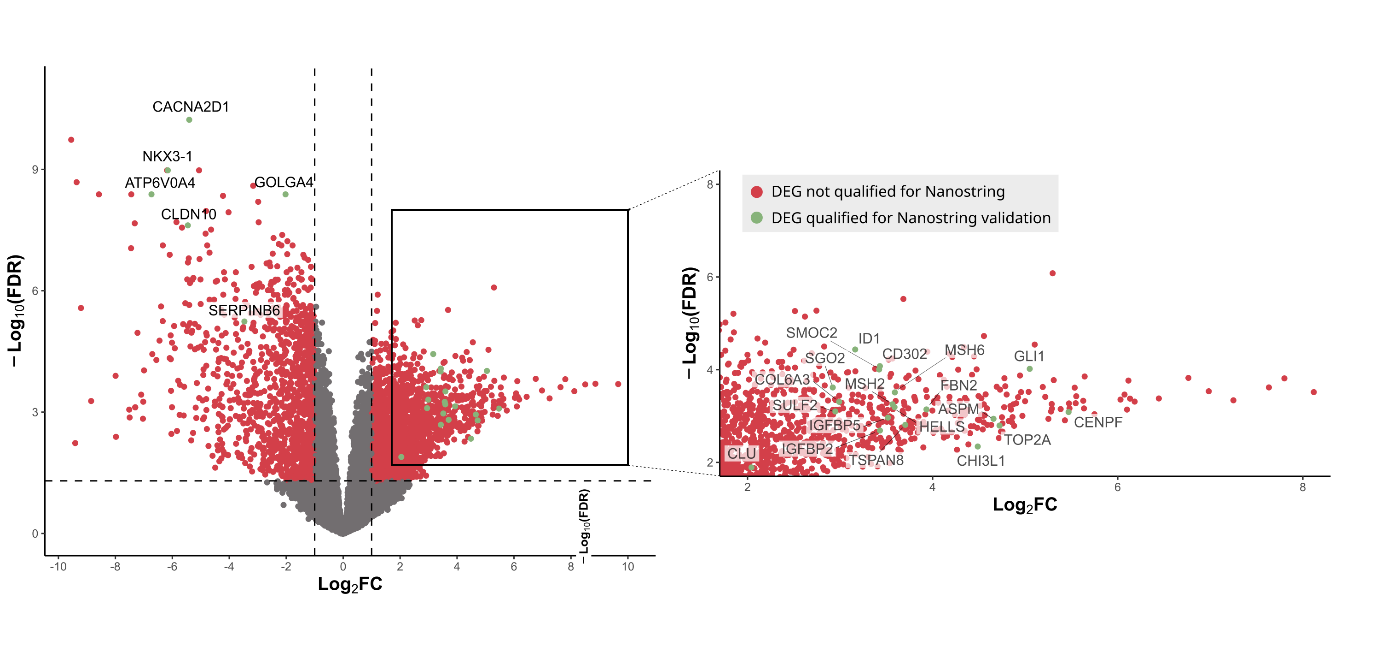


**Supplementary Figure 2.** **Unfiltered differentially expressed genes (DEGs) between prostate cancer (PC) and benign prostate hyperplasia (BPH).** Volcano plot showing 2923 differentially expressed genes (DEGs) in PC vs. BPH (-1>log_2_FC>1, FDR>0.05) out of 12 185 identified genes. After additional filtering (mean normalized counts per million (CPM) >10 in each group) DEGs in green were selected for validation by targeted mRNA profiling (NanoString technology) based on the highest differences in expression and presence of mRNA in both samples (PC and BPH). For clarity, an enlarged section of the volcano plot shows the DEGs upregulated in PC.

**A)**

**
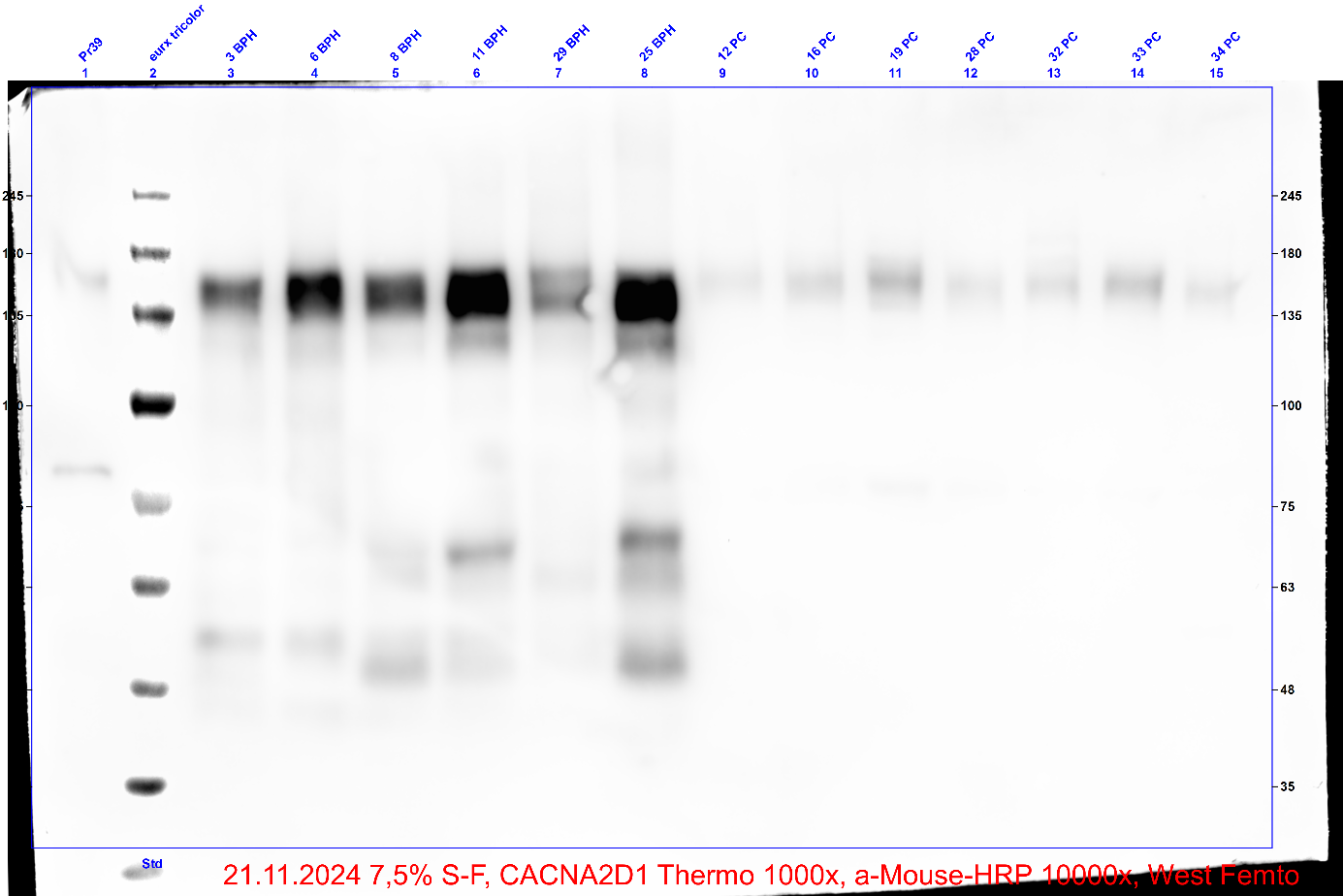

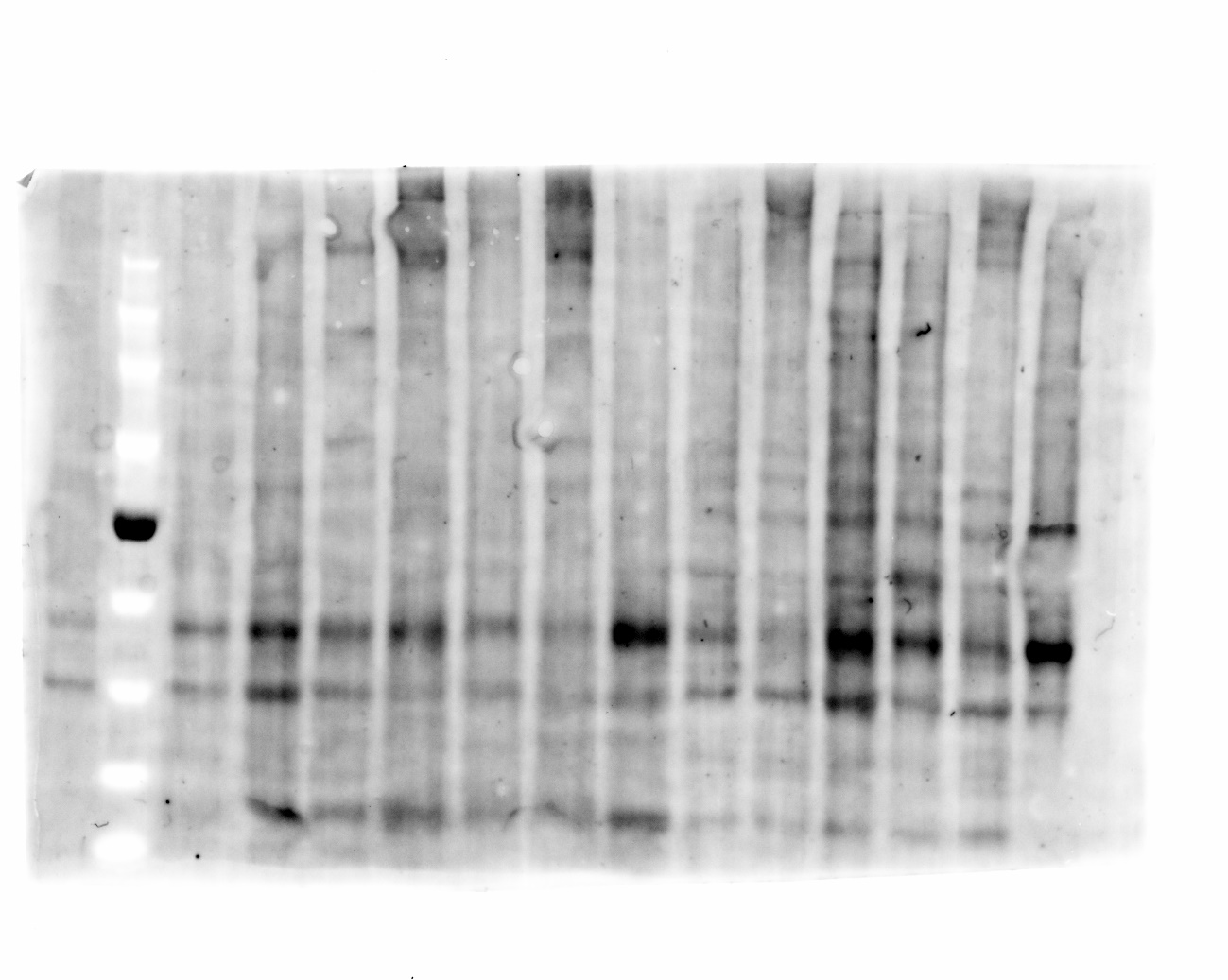
B)**

**C)**

**
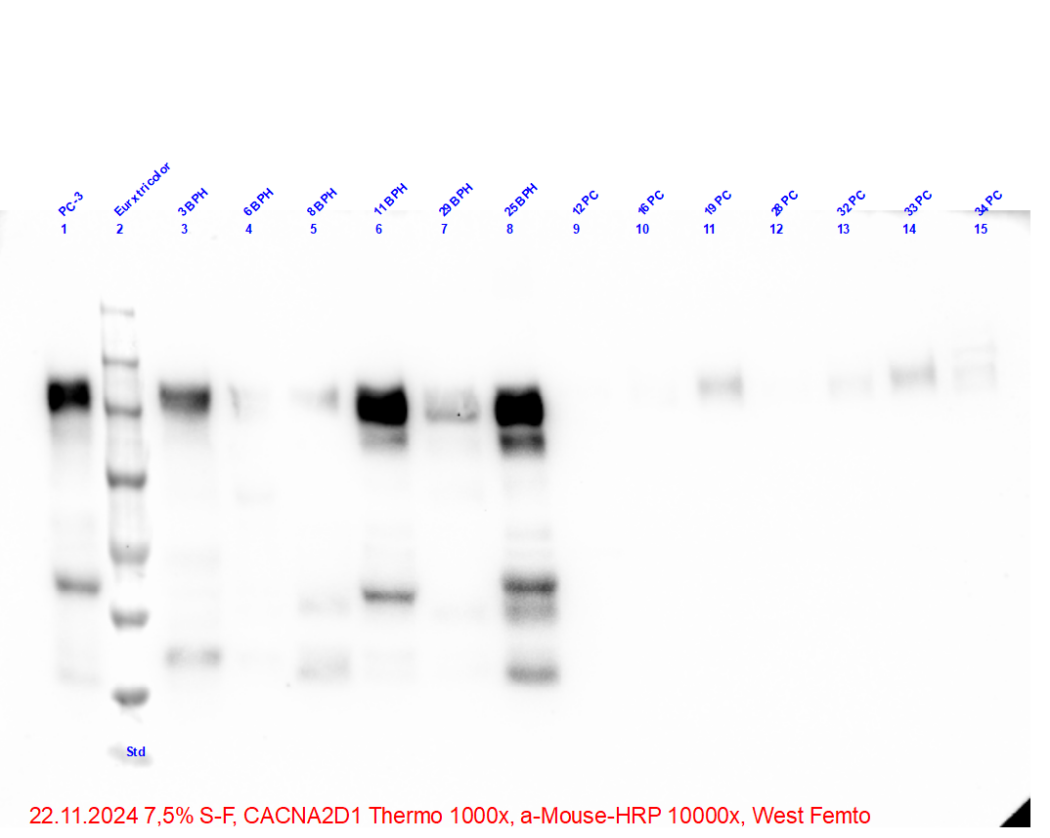
**

**
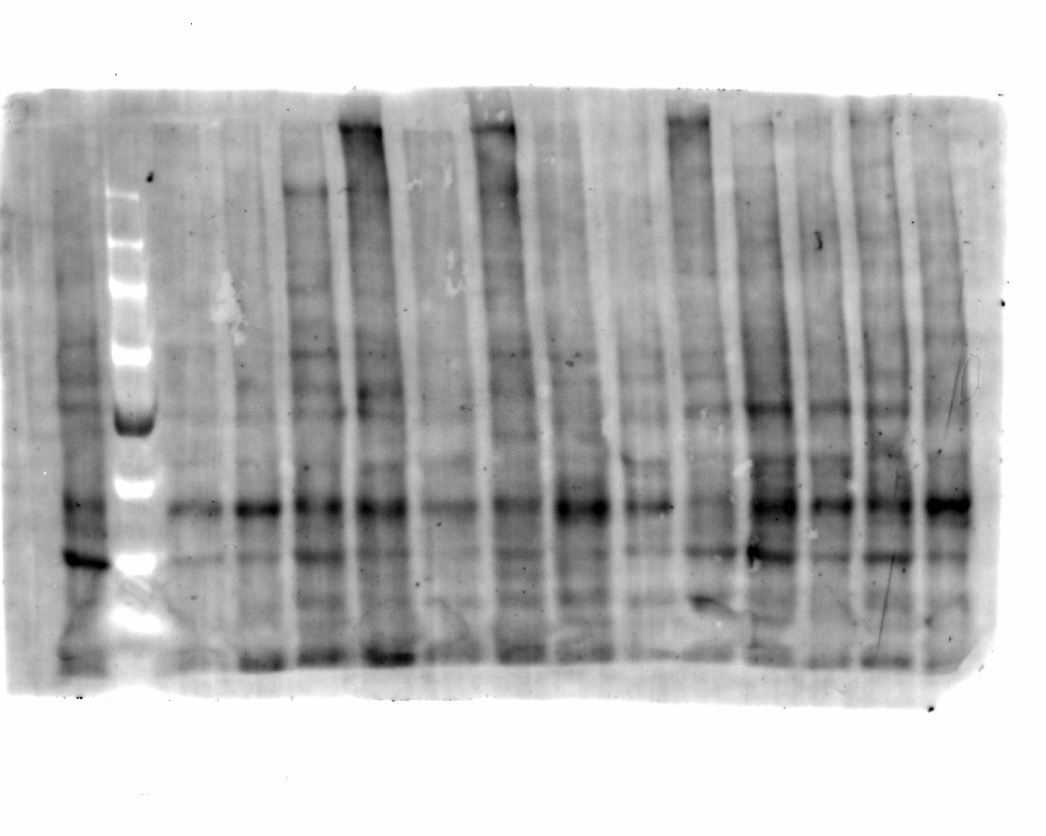
**

**Supplementary Figure 3. A) Full-length blot presented in Fig.3A, and B) corresponding Stain-Free loading control. C) Blot replicate used together with A) for densitometric analysis presented in Fig. 3B. and D) corresponding Stain-Free loading control.**
